# Rhinoscleroma with Pharyngolaryngeal Involvement Caused by* Klebsiella ozaenae*

**DOI:** 10.1155/2016/6536275

**Published:** 2016-05-12

**Authors:** J. Gonzales Zamora, A. R. Murali

**Affiliations:** ^1^Division of Infectious Diseases, Augusta University, Augusta, GA 30912, USA; ^2^Department of Gastroenterology, University of Iowa Hospital and Clinics, Iowa City, IA 52242, USA

## Abstract

Rhinoscleroma is a chronic, slowly progressive granulomatous bacterial infection that is endemic to the tropical world, namely, Central America and Africa. It is occasionally seen in the United States of America (USA). It predominately affects the nasal mucosa but can also involve the rest of the upper respiratory tract. The well-known causative agent for rhinoscleroma is the bacterium* Klebsiella rhinoscleromatis*, a subspecies of* Klebsiella pneumoniae*. However,* Klebsiella ozaenae* can also, albeit very rarely, cause rhinoscleroma. The diagnosis is confirmed by histopathology examination that shows the characteristic Mikulicz cells, considered pathognomonic for this infection. We report a patient with histologically proven rhinoscleroma with pharyngolaryngeal involvement in whom cultures yielded* Klebsiella ozaenae*. To the best of our knowledge, only two cases of rhinoscleroma due to* Klebsiella ozaenae* have been reported in the literature to date. Our case illustrates the importance of recognizing this infection in a nonendemic setting such as the USA. A lack of awareness and a delay in the diagnosis of this disease can lead to complications including upper airway obstruction, physical deformity, and, rarely, sepsis. In addition, it must be remembered that the treatment of rhinoscleroma is challenging and requires a prolonged course of antibiotics to achieve a definite cure and avoid relapses.

## 1. Introduction

Rhinoscleroma is a chronic granulomatous infection that affects the upper respiratory tract from the nose down to the trachea. This disease is found primarily in impoverished areas of the Middle East, Eastern Europe, Africa, and Central and South America. It is reported uncommonly in the USA, where cases are seen in immigrants from endemic countries. The bacterium implicated as the causative agent of this infection is* Klebsiella rhinoscleromatis*. However,* Klebsiella ozaenae*, which is typically associated with primary atrophic rhinitis, has also been reported as a cause of rhinoscleroma in only 2 previous case reports. Rhinoscleroma predominantly affects the nose; other areas of the respiratory tract such as the larynx or oropharynx are affected in a minority of patients. We describe a rare case of rhinoscleroma with pharyngolaryngeal involvement due to* Klebsiella ozaenae* in a patient hailing from Mexico.

## 2. Case Report

**Figure 1:**
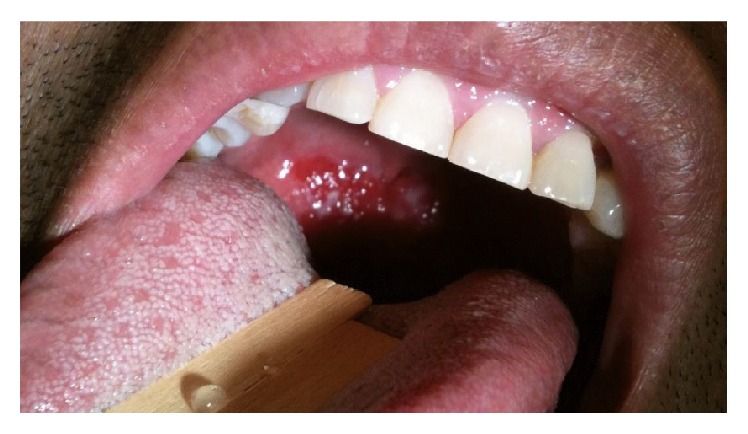
Erythematous tissue with white exudates covering the oropharynx.

A 47-year-old male originally from Mexico presented to our hospital with persistent nasal congestion, sore throat, hoarseness of voice, and dysphagia. His symptoms first started 23 years before while in Mexico. At that time, he reported having had multiple episodes of epistaxis and the presence of an “extra skin” in his nose. He also reported that he had a surgical procedure at initial presentation in which his “nostrils were scraped” and subsequently was put on antibiotics for 6 months (patient unaware of the name of the antibiotics he received). His symptoms improved, only to resurface ten years later. Shortly thereafter, he moved to the USA. He experienced progressive worsening of nasal congestion, difficulty swallowing, and hoarseness of voice and went to a primary care physician for evaluation. He received 3 doses of intramuscular ceftriaxone followed by oral antibiotics. He reported a mild clinical improvement followed by rapid relapse with continued symptoms progression. He presented to our hospital for further evaluation. By the time of hospital presentation, in addition to the above symptoms the patient also noted a mass protruding into the back of his throat. He denied weight loss, fever, or chills. His past medical history was significant for treated pulmonary tuberculosis at the age of 16. He denied any cigarette smoking, drug use, or alcohol intake. His family history revealed that his maternal uncle and his oldest son had constant rhinorrhea and his mother had a mass in her nose, but all affected family members remained in Mexico with limited access to medical care. Physical exam showed an erythematous, irregular soft tissue lesion covering the entire posterior oropharynx with some white exudate (Figure 1). The mucosa of the nares had a similar appearance, but no discreet mass was appreciated. The remainder of the exam was unremarkable.

**Figure 2:**
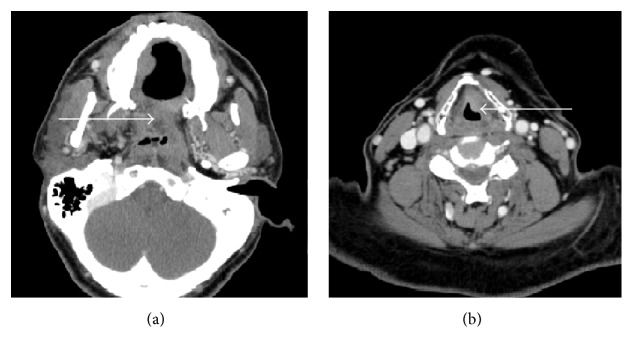
(a) 21 × 24 × 25 mm mass involving the soft palate significantly narrowing the nasopharynx. (b) 15 × 9 mm lesion along the anterior aspect of the left false vocal cord.

Computed tomography of the neck showed a 21 × 24 × 25 mm mass in the soft palate narrowing the nasopharynx (Figure 2(a)) and a lesion of 15 × 9 mm in the anterior aspect of the left false vocal cord (Figure 2(b)). There was also an asymmetric thickening of the left tonsil. No cervical adenopathy was detected. An esophagogram did not reveal any evidence of obstruction.

**Figure 3:**
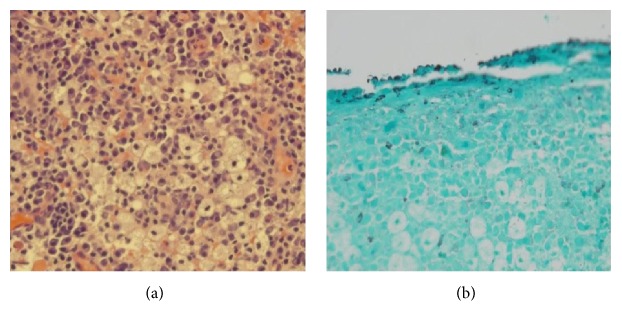
(a) Histopathology showing plasma cell infiltrate mixed with foamy macrophages, known as Mikulicz cells (H&E, 40x). (b) Coccobacilli seen inside the macrophages (GMS, 40x).

Otolaryngology was consulted and performed biopsies of the oropharyngeal mass and the left tonsil. Tissue was submitted for pathology and microbiology studies. Histopathology with hematoxylin and eosin (H&E) stain revealed plasma cell infiltrates mixed with foamy macrophages, known as Mikulicz cells (Figure 3(a)). These findings were consistent with rhinoscleroma. Gomori methenamine silver (GMS) stain was also performed and showed intracellular coccobacilli (Figure 3(b)). Tissue cultures yielded* Klebsiella ozaenae*, resistant only to ampicillin. The automated system used to identify this isolate was the MicroScan Walkaway 96 (Siemens, Sacramento, California).

The patient was started on trimethoprim-sulfamethoxazole but developed body aches and cramps as side effects. Therapy was subsequently changed to ciprofloxacin and doxycycline, planning to complete a six-to-twelve-month course. Unfortunately, he was subsequently lost to follow-up.

## 3. Discussion

Rhinoscleroma is a chronic granulomatous disease of the upper respiratory tract caused by* Klebsiella rhinoscleromatis*. This infection was first described in 1870 by Von Hebra [1]; seven years later, Mikulicz described the histology and the characteristic foam cells that bear his name [2]. In 1882, Von Frisch [3] identified the causal agent now known as* Klebsiella rhinoscleromatis*. This infection is prevalent in rural areas with poor sanitation and is considered endemic in Africa, Southeast Asia, and Central and South America. Although most cases occur in developing countries, recent immigration patterns have led to an increasing number of patients with rhinoscleroma in the USA [4].

The mode of transmission is not well understood, but it is probably through contaminated airborne particles, which are expelled by coughing and sneezing or by contact with fomites. An epidemiologic study that included 11 cases published by De Pontual et al. found that 3 out of 11 patients had a significant family history of rhinoscleroma [5]. These findings suggest that transmission could be secondary to prolonged household contact with infected family members. A genetic predisposition is also possible since mutations affecting genes that encode components of S-nitrosoglutathione reductase (GSNOR) have been found to increase the susceptibility to* Klebsiella pneumonia* infection in mice [6]. The haplotype HLA-DQA1^*∗*^03011-DQB^*∗*^0301 has been another important factor associated with the development of this disease [7]. Of note, our patient had 3 relatives with a presumptive history of rhinoscleroma.

This infection is typically divided into three stages: catarrhal, granulomatous, and fibrotic. The catarrhal stage causes symptoms of nonspecific rhinitis that can last for weeks to months and often evolves into purulent and fetid rhinorrhea with crusting. The granulomatous stage results in the development of a bluish red nasal mucosa and intranasal rubbery nodules or polyps. Epistaxis, nasal deformity, and destruction of the nasal cartilage may also be noted (Hebra nose). The third stage is characterized by extensive fibrosis leading to extensive scarring and possible nasal/laryngeal stenosis.

The areas of the respiratory tract that are typically involved are the nasal cavity and nasopharynx. This infection may also affect the larynx, trachea, bronchi, the middle ear, oral cavity, paranasal sinuses, orbit, and soft tissues of the lips. Fawaz and colleagues published one of the largest cases series of rhinoscleroma in Egypt, which included 88 patients. In this review, the nose was compromised in 100% of the cases. Pharyngeal rhinoscleroma was found in only 9 (10%) patients, whereas pharyngolaryngeal involvement occurred in 19 (21.5%) cases. Involvement of the trachea was only reported in 1 (1%) case [8]. Our patient had involvement of multiple sites that included the nose, soft palate, and false vocal cords, which is a relatively uncommon manifestation of this disease.

The diagnosis is usually confirmed with histopathology revealing the characteristic Mikulicz cells, Russel bodies, and positive Warthin-Starry stains. It is typically described that routine cultures are positive for* Klebsiella rhinoscleromatis* in only 50%–60% of patients in the granulomatous stage [9]. However, if the biopsy is repeated two or three times the yield of the culture can reach 100% [10].

In our patient, the biopsy culture grew* Klebsiella ozaenae*, which is not a typical organism associated with rhinoscleroma. This species is the causative agent of ozena, which is a chronic rhinitis characterized by atrophic changes in the nasal mucosa and turbinates, enlargement of the nasal passages, and a foul-smelling mucopurulent discharge that tends to dry into crusts. Unlike* Klebsiella rhinoscleromatis*,* Klebsiella ozaenae* has been described in clinical diseases other than chronic rhinitis such as meningitis, cerebral abscess, bacteremia, and pyogenic hepatic abscess [11–13].

Microbiologic studies using biochemical tests are very accurate in differentiating the subspecies of* Klebsiella*. Our laboratory used the MicroScan Walkaway 96 (Siemens, Sacramento, California) which has an accuracy of 99% for identification of fermentative Gram-negative organisms. From these data, we think that the subspecies* Klebsiella* was properly identified as* ozaenae* from our patient's cultures.

There are only 2 cases reported in the literature of* Klebsiella ozaenae* causing rhinoscleroma. The first case was reported by De Champs et al., who described an Algerian patient with a 4-year history of dysphonia that was found to have a lesion in the front third of the right vocal cord. Histopathological examination was consistent with laryngeal scleroma. Her nasal and pharyngeal cultures grew* Klebsiella ozaenae* along with* Morganella morganii*,* Pseudomonas aeruginosa*, and alpha-hemolytic streptococci [14]. The second case was reported by Costa Climent et al., who described the case of a Romanian male presenting with a two-month history of dyspnea, dysphonia, and abundant purulent, foul-smelling nasal secretions. Bronchoscopy revealed inflammation of both vocal cords and the subglottic region and white papillary lesions with black crusts and green secretions throughout the entire length of the trachea. The pathology findings of the lesions were of a mixed inflammatory infiltrate containing abundant bacteria. The cultures of nasal biopsies, bronchoalveolar lavage fluid, and bronchial aspirate yielded* Klebsiella ozaenae*. This last case was considered as laryngotracheal rhinoscleroma based on clinical and macroscopic findings; however the histopathology was not reported to have the typical Mikulicz cells or Russel bodies [15]. It is interesting that these two cases, like our patient, presented involvement of the larynx which is an uncommon manifestation of this disease.

The pathogenesis of rhinoscleroma remains poorly understood primarily due to the lack of characterized* in vitro* and* in vivo* models. Very little is known about the mechanisms underlying this condition; however Mikulicz cells, considered the hallmark of rhinoscleroma, may play a fundamental role in the development of chronic granulomatous inflammation observed in this infection [16]. As a matter of fact, Fevre and colleges conducted a study aimed at characterizing the formation of Mikulicz cells in a mouse model. Their data showed that interleukin-10 is highly expressed upon infection with* Klebsiella rhinoscleromatis* and suggested an important role of this cytokine in the phenotypic maturation of Mikulicz cells and thereby in rhinoscleroma pathogenesis [16]. Other authors have developed murine models to explore the pathophysiological mechanism of* Klebsiella ozaenae* infection and have found a significant increase in the levels of certain cytokines such as MIP-2, KC, and IL-6 [17].

The treatment of both rhinoscleroma and ozena involves antibiotics coupled with surgical debridement in cases of airway obstruction or cosmetic deformity.* Klebsiella rhinoscleromatis* is inhibited* in vitro* by the majority of antibiotics that are active against Gram-negative bacteria with the exception of penicillin and ampicillin [18]. However,* in vivo*, the antibiotics that have shown activity include streptomycin, doxycycline, tetracycline, rifampin, second- and third-generation cephalosporins, sulfonamides, clofazimine, ciprofloxacin, and ofloxacin [19]. Which antibiotic is the best to treat this infection is not known with certainty, as no randomized clinical trials have been conducted to assess the efficacy of each antimicrobial agent.* Klebsiella rhinoscleromatis* is an intracellular bacterium, so theoretically it responds best to antibiotics that can achieve high concentrations in macrophages. Fawaz et al. recommended in their study the use of rifampin, trimethoprim-sulfamethoxazole, or ciprofloxacin for at least 2-3 months [8]. Dual antibiotic therapy has also been proposed in some case reports. Suchanova and colleges suggested treatment with ciprofloxacin and cotrimoxazole [20], whereas Tan and colleges achieved good results with a combination of ciprofloxacin and doxycycline [9]. All the studies concur that antibiotic treatment should be prolonged. The recommended duration of therapy varies from 2 months to 1 year.

One of the main difficulties in treating rhinoscleroma is the high rate of disease relapse, reaching 26% in some case series. According to Fawaz et al., such a high rate of recurrence may be attributed to noncompliance, short duration of therapy, extensiveness of the lesions, or progression to a cicatricial stage, which has poor response to medications due to poor vascularity in the affected tissues [8].

In regard to* Klebsiella ozaenae*, it is traditionally susceptible to multiple antibiotics such as tetracyclines, cephalosporins, aminoglycosides, quinolones, and chloramphenicol. This organism, unlike* Klebsiella rhinoscleromatis*, is usually susceptible to ampicillin, except in cases of prior antibiotic exposure, in which the susceptibility rate is only 26% [11]. Our patient's isolate was resistant to ampicillin, which was expected, given the multiple antibiotic regimens he had received prior to his presentation to our hospital.

Regarding the treatment of the two cases of rhinoscleroma caused by* Klebsiella ozaenae*, Costa Climent and colleagues treated their patient initially with ciprofloxacin and cefuroxime for 2 months. Therapy was then changed to trimethoprim-sulfamethoxazole and inhaled tobramycin for 1 month. According to the report, treatment with trimethoprim-sulfamethoxazole was maintained until cultures became negative; however the total duration of therapy was not specified [15]. In the case reported by De Champs et al., the patient was treated with cefixime for 3 weeks [14].

Our patient was treated initially with trimethoprim-sulfamethoxazole; however, due to side effects, the treatment regimen was changed to ciprofloxacin and doxycycline. Unfortunately, he was lost to follow-up and response to this regimen could not be assessed.
